# Contextualizing the ecology of plant–plant interactions and constructive networks

**DOI:** 10.1093/aobpla/plad035

**Published:** 2023-07-01

**Authors:** Gianalberto Losapio

**Affiliations:** Faculty of Geosciences and Environment, Institute of Earth Surface Dynamics, University of Lausanne, UNIL Mouline, 1015, VD, Switzerland; Department of Biosciences, University of Milan, Via Celoria 26, 20133, Milan, Italy

**Keywords:** Biodiversity, community ecology, ecological networks, plant facilitation, species interactions

## Abstract

Botanical concepts have traditionally viewed the environment as a static box containing plants. In this box, plants compete with one another and act as passive resource consumers subjected to the environment in a top-down manner. This entails that plants have only negative effects on other plants and have no influence on the environment. By contrast, there is increasing evidence that plants have positive, bottom-up engineering effects and diversity effects on other plants and on the environment. Here, to overcome the limitations of top-down environmental control, antagonistic-only and pairwise interactions, I propose the concept of constructive networks. Constructive networks unify niche construction and network theory recognizing that (i) plants have manifold ecological functions and impacts on their neighbours, and (ii) the environment shapes and is shaped by diverse organisms, primarily plants. Constructive networks integrate both plant–environment and plant–plant interactions in a relational context. They address how plants influence the environment and support or inhibit other plant species by physically, biochemically and ecologically shaping environmental conditions. Constructive networks acknowledge the fact that diverse plants change and create novel environmental conditions and co-produce, share and transform resources, thereby influencing biological communities and the environment in constructive ways. Different interaction types are considered simultaneously in constructive networks. Yet, the main limitation to understanding constructive networks is the identification of plant links. This barrier may be overcome by applying complexity theory and statistical mechanics to comparative data and experimental field botany. Considering multiple interaction types and feedback between plants and the environment may improve our understanding of mechanisms responsible for biodiversity maintenance and help us to better anticipate the response of plant systems to global change.

## Introduction

For centuries, scientists deduced properties of natural systems and hence inferred all their possible past and future states by breaking them up into basic units and meticulously measuring each and every part in isolation. This reductionist approach works well with inanimate bodies, but poses serious limits when it comes to understanding living systems (Prigogine and Stengers 1979).

The common feature of living systems is that they involve many ‘components’ that interact with each other and with their environment in a non-linear way, and are consequently organized in an integrated, emergent ensemble (Ulanowicz 2018). Hence, each component influences the others and it is also influenced by them. By treating organisms, plant communities or ecosystems like single isolated genes, cells, individuals or populations, we disregard their functionality and their interactive nature (Kauffman 2019). As a consequence, plant systems cannot be fully understood by analysing parts of them in isolation.

With the main goal of studying the relationships underlying biological systems, ecology has developed as the science of ‘how organisms interact with each other and with their environment’ (Bersier 2007; Levin 2009). Focusing on system mechanisms, mass and energy exchanges, and species interactions, ecology emphasizes relationships and processes over objects. This way, plant ecology can overcome limitations posed by biological reductionism and address the complexity of plant systems.

Ecological thinking in botany can be traced back to the ancient philosopher Theophrastus (371–287 BC), who classified plants according to their reproduction, locality, size and practical uses in his *Historia Plantarum*. But the scientific field of plant ecology emerged from its biogeographic origins during the 19th century, thanks to the work of the naturalist Alexander von Humboldt (1769–1859), who first studied how the form and function of plants are affected by physical conditions, and provided the first description of global vegetation distribution according to climate (von Humboldt and Bonpland 1805). Since then, plant ecology has grown from describing patterns of species and communities to inferring processes driving species diversity and diversification, and is now moving towards the studies of species–environment interactions and the role of plant interactions for the functioning and stability of global ecosystems.

It is well recognized that the history of biodiversity is fundamentally a history of species interactions (Thompson 1999; Bascompte and Jordano 2014). In this sense, biodiversity is more than a list of genes or species. Solid evidence indicates that positive interactions are widespread in nature as mutualism and facilitation are increasingly recognized to be fundamental processes in the ecology and evolution of plants (Stachowicz 2001; Callaway et al. 2002; Bruno et al. 2003; Callaway 2007; Bascompte and Jordano 2014; Cavieres et al. 2014; Losapio et al. 2021b). Despite recent advances in analysing networks of interactions involving plant species (Levine et al. 2017; Alcántara et al. 2019; Losapio et al. 2021b), we are still far from understanding plant–plant networks and their role in maintaining biodiversity and regulating ecosystem functioning.

## The problem with analogies from animal studies

**Figure 1:**
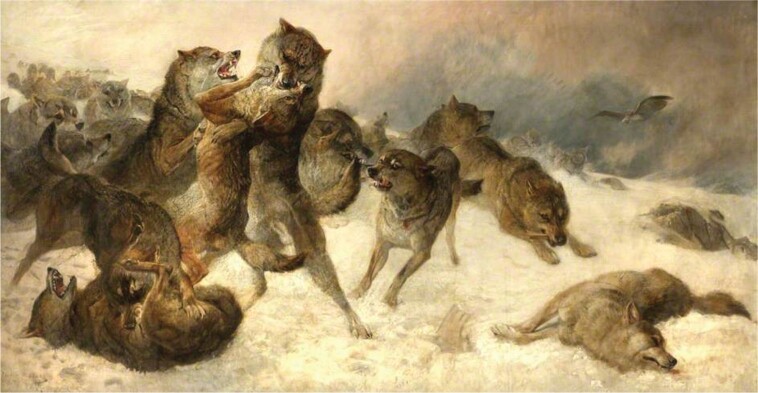
The struggle for existence, George Bouverie Goddard 1879 (1832–86). Courtesy National Museums Liverpool. Photo credit: Walker Art Gallery.

The idea of nature and life as a competitive race dates back to the late 18th century, and it is widespread not only in natural sciences but it is also embedded in many areas of social sciences and the arts. This idea is illustrated well by the painting *The struggle for existence* (1879) (Fig. 1), which depicts the typical human view of a nature ‘red in tooth and claw’. This painting shows the degree to which culture and sciences have been dominated by antagonistic-oriented paradigms for centuries.

**Figure 2:**
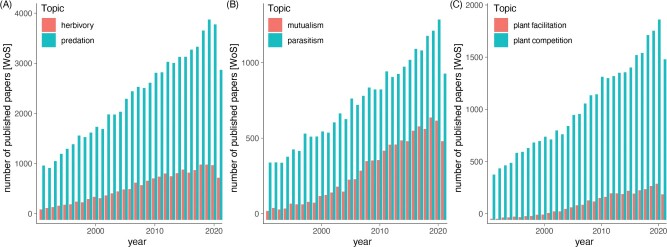
Number of papers published from 1992 to 2022 as indexed on Web of Science (data retrieved on 16 December 2022) focusing on animal and antagonistic interactions over plant and positive interactions. Search terms (title and abstract) were herbivory, predation, mutualism, parasitism, plant competition and plant facilitation.

Such a strong focus on animal antagonistic interactions is exemplified by the emphasis on predation over herbivory (Fig. 2A) and parasitism over mutualism (Fig. 2B): during the last 30 years (from 1992 to 2022), studies on predation or parasitism have been published 3–4 times more than studies on herbivory or mutualism. If we consider that plants constitute 95 % of terrestrial biomass (Bar-On et al. 2018), then this disproportionate focus on animal antagonistic interactions is especially striking. Given the central role attributed to competition and predation between animals, it is not surprising that theoretical and experimental studies in botany and plant ecology have been centred around antagonistic interactions among plants (Bronstein 2009). Indeed, studies on plant competition are published 10 times (!) more than those on plant facilitation (Fig. 2C).

Despite half a century of research on competitive interactions and more than 33 000 papers published on plant competition over the last 30 years, it is not clear how different species co-exist in natural communities (Verhoef and Morin 2010; Saavedra et al. 2017) nor how plant diversity supports ecosystem functioning (Wright et al. 2017). Most likely, the majority of these papers assumed competition as a default explanation. Major focus on predation and competition left out of the picture fundamental biological phenomena such as mutualism in plant–animal interactions and facilitation in plant–plant interactions.

Unfortunately, the study of interactions between plant species has developed around theories and models formalized for antagonistic interactions between animals, an approach that poses serious limitations in understanding the ecology and evolution of plants. There are four fundamentals of plant biology that invalidate zoocentric analogies, assumptions, theories and explanations. These four intrinsic properties of plants make them interconnected and interdependent and coupled to their environment.

First, zoocentric models of predator–prey or consumer–resource interactions assume plants as consumers. On the contrary, plants are the producers. Plants do not (only) consume nutrients, but first and foremost they produce organic matter by converting solar energy. In natural communities, that is, not in agricultural settings where biomass is exported from the system, matter (i.e. resources and nutrients) is recycled within the ecosystem through plant biogeochemical paths, species interactions (e.g. herbivory) and environmental disturbance (e.g. fire) (Keddy 2017). Notably, thanks to microorganism mutualistic partners hosted in their roots, plants increase soil resources by transforming mineral compounds into organic nutrients (Tedersoo et al. 2020). Most importantly, while predation and herbivory are phenomenologically similar, animal prey cannot benefit from predation, whereas many plants may benefit from herbivory, such as in the case of compensatory growth (Archibald et al. 2019).

Second, the concept of the individual in plants is remarkably different from the concept of the individual in the animal kingdom as plants are not individualistic but modular organisms (Keddy 2017). What is an individual plant? How do we deal with clonal plants for which the concept of ‘individual’ is much more blurred? Is an individual a ramet or a genet? The notorious example of the Pando quaking aspen (*Populus tremuloides*) clonal colony of an ‘individual’ constituting a 100-acre forest in Utah (USA) is emblematic of the difficulties associated with defining and identifying individual identity in the plant kingdom (Mitton & Grant 1996). Annual plants are the exception as their life cycle and individual fitness components are comparable to those of animals (Levine et al. 2017), but annual plants are poorly representative of global flora and biomes, making them unsuited to broader generalization and deeper understanding. For these reasons, zoocentric models based on individual fitness have a limited validity when applied to plant diversity and plant communities.

Third, plants have no consciousness or cognition. Plants do not deliberately or intentionally make decisions to facilitate other plants. Although plants form communities, communicate and actively respond to cues and their changing environment, they do not display social behaviour, nor do they have intentions to co-exist in the same community or consciously choose their mating partners (Mescher and Pearse 2016). Nevertheless, teleology, anthropomorphism, the figurative and metaphoric character of language and our semantic interpretations (Mescher and Pearse 2016; Varella 2018) embedded in animal–plant analogies may create bias, misunderstanding and misinterpretation in plant science.

Finally, putting too much emphasis on the sessile nature of plants brings along a series of limitations. One is the prominent emphasis on deterministic top-down control of the environment over plants. Accordingly, plant species and ecological communities are analysed as a typological construct— assemblages of populations or species that share common adaptations and differentiate niches in response to levels of competition (Callaway 2007; Saavedra et al. 2017). But this view of niches and the environment as an ‘abiotic’ static box falls short of explaining the fact that organisms can actively modify their environment by creating and altering biophysical conditions (Lewontin 1983). As a matter of fact, organisms are not just passively influenced by abiotic factors, but rather they can ‘act’ upon their surroundings and change the environment (Chase and Leibold 2003). Unfortunately, the constructive effects of plants on the environment hardly emerge in controlled greenhouse conditions.

A second limitation posed by the focus on plants as immobile organisms is related to the fact that, with few exceptions limited to commensalism and parasitism, we cannot see plant–plant interactions with the naked eye or under a microscope. It is much easier to look at ‘who eats whom’ or ‘who is visited by whom’ than ‘who facilitates whom’, simply because the latter is usually not apparent to us. We cannot observe a plant facilitating another plant in the field, as discussed below.

These four fundamental differences may explain why zoocentric ecologists and evolutionary biologists refute the idea that species interaction outcomes are flipped in plant communities: while competition among animals increases when resources are scarce, plant competition increases with increasing resources, such as in agricultural systems (Keddy 2017; Schöb et al. 2018), whereas plant facilitation prevails in harsh and poor-resource environments (Callaway et al. 2002).

## Plant–plant interactions beyond competition

As much as the environment influences plants, plants can modify their surrounding environment by creating new habitats and altering former ones. Thus, plants ultimately influence the dynamic of evolutionary and ecological processes in fundamental ways (Kéfi et al. 2012). The implications of these facts are twofold. First, plant species are not only selected by the environment top-down, but can rather influence and change environmental conditions bottom-up (Jones et al. 1994; Ellison et al. 2005; Schöb et al. 2012; Losapio et al. 2023). Second, plants can create environmental conditions that allow other species to thrive, that is, facilitate other plants (Bruno et al. 2003; Callaway 2007; McIntire and Fajardo 2014).

Plant facilitation is the positive interaction between two or more plant species in which one plant benefits from another while the other plant may or may not benefit from the relationship (Bertness and Callaway 1994; Callaway 2007). Facilitation occurs if the overall improvement of the environment results in a positive net outcome for at least one plant species. There is facilitation when plants or different species are experiencing greater dispersal success, recruitment, growth, survival, reproduction and fitness in the presence of neighbours than in their absence (Callaway 2007). The difference between mutualism and facilitation is that, in mutualism, both partners benefit from the interactions, whereas in facilitation, the at least one partner benefits while the other one may not. For example, annual herbs in arid ecosystems establish, recruit and survive more often, make better photosynthesis, and produce more seeds beneath a canopy of shrubs than in shrub absence (Pugnaire 2010). In turn, the effects for the shrub may range from negative to positive, including neutral, depending on the ecological process considered and the climate (Schöb et al. 2014; Losapio et al. 2021a). Hence, facilitation has usually been seen as commensalism (Callaway 2007), but it may cover the whole spectrum from mutualism to parasitism (Schöb et al. 2014).

Across different systems, from mountains to deserts and kelp forests, facilitation mechanisms by plants are mainly due to the modification of local environmental conditions (Bruno et al. 2003; Callaway 2007; McIntire and Fajardo 2014) and the construction of novel niche space (Schöb et al. 2012) in a way that benefits other species. This facilitation process includes the following mechanisms (for a complete discussion, see Callaway, 2007; McIntire and Fajardo 2014): (i) creation of habitat structural features and shelter, e.g. by providing growth substrate or physical protection against herbivores; (ii) increase of resource availability, e.g. by improving soil organic matter, providing nutrients, increasing soil moisture or attracting pollinators; and (iii) decrease of stress and disturbance, e.g. by lowering UV radiation, vapour pressure deficit and pathogen incidence, stabilizing soil and decreasing temperature extremes.

As opposed to mutualism, facilitation between two plant species involves both direct effects (e.g. physical presence as well as the effects of plant activity on the environment) and indirect interactions (e.g. involvement of species from different trophic levels such as pollinators, herbivores or microorganisms) (Callaway 2007). Regardless of the outcome, mechanism-specific benefits and costs of plant facilitation may occur at the same time (Losapio et al. 2019). This is the case, for instance, when facilitation for recruitment and vegetative growth goes along with competition for pollination or seed dispersal (Ghazoul 2006; Rumeu et al. 2019; Losapio et al. 2021a). A final difference between facilitation and mutualism lies in the research approach they received in the last two decades. While mutualistic interactions have undergone the ‘network revolution’, plant facilitation has been analysed by looking at pairwise interactions, as the study of facilitative interactions has hardly considered ecological networks (Losapio et al. 2019).

## Ecological networks involving plant facilitation

Since the end of the 20th century, many systems including the human brain, food webs, financial markets, and electrical grids, among others, have been described as networks (Cohen and Havlin 2010). These networks, mathematically modelled as graphs, are defined by nodes that are connected through links. The generality and flexibility of such mathematical tools allowed scientists from multiple fields to reveal universal patterns and processes across diverse systems (Newman et al. 2006). The study of food webs, implemented by analysing the network of ‘who eats whom’, greatly improved our understanding of the complexity and stability of trophic interactions among species, providing important insight into the persistence and dynamics of natural ecosystems. For instance, now we can better predict and anticipate the impact of global change on food webs (Cohen and Havlin 2010) and manage ecosystems accordingly. Certainly, network thinking is by no means new to ecology (Bascompte and Jordano 2014). Darwin was among the first to recognize the importance of ecological networks when he described natural communities as a ‘tangled bank of complex species interactions’ (Bersier 2007). Thanks to the recent confluence of ecological and network sciences, a number of new opportunities for approaching plants from a complex systems perspective are now open.

Research on mutualistic networks involving plant–pollinator interactions and seed dispersal has shed light on ecological and evolutionary processes maintaining biodiversity at the community level (Bascompte and Jordano 2014). Plant and pollinator communities are composed by heterogeneous interactions differentiated along a gradient of specialization–generalization. The majority of species are specialists that interact with only a few other species, while the minority is composed of generalist species. Understanding this particular arrangement of network-level interactions is important for the maintenance of biodiversity given that the particular structure of ecological networks has important implications for biodiversity dynamics, particularly for the stable coexistence of species and the robustness of ecosystems (Bascompte and Jordano 2014).

Yet, networks of interactions within plant communities have been less explored in comparison to other ecological systems. On the one hand, theoretical models of perfectly intransitive competitive networks showed that coexistence via intransitive competition (e.g. species A outcompetes species B, B outcompetes C, and C, in turn, outcompetes A) is a stabilizing niche mechanism that might favour species diversity (Grilli et al. 2017). On the other hand, empirical models of community-level facilitation showed recurrent patterns underlying the structure of plant networks between facilitator and facilitated species (Verdú and Valiente-Banuet 2008; Saiz et al. 2018; Alcántara et al. 2019; Losapio et al. 2019). These plant facilitation networks can either be organized in a nested way around a core of overlapping interactions, or in a modular way with independent groups of species. Either way, plant facilitation networks showed high resistance to environmental change drivers related to stress (Losapio et al. 2019). This resistance to external perturbations can decrease local coextinctions, thus sustaining biodiversity. Looking at different interaction types, it turned out that biodiversity increases with increasing prevalence of network motifs that include both facilitation and competition among plants (Losapio et al. 2021b).

Despite these recent advances, we are still far from building real-world comprehensive and robust networks of interactions among plant species, which also hinders our ability to unveil factors responsible for predicting community structure and dynamics.

## Constructive networks

On top of limitations arising from zoocentric models, there is an additional issue with understanding plant–plant interactions at the network level. With increasing plant diversity, it becomes difficult to experimentally and computationally assess all possible combinations of species interactions. For instance, a community of only 10 plant species would require parameterizing all possible combinations of intra-specific (${n}=10$), two-species (${n}=45$), three-species (${n}=120$), four-species (${n}=210$) etc. interactions, which is practically unfeasible.

All together, identifying all possible facilitative or competitive interactions in a diverse community is not as straightforward because experimental manipulation is often unfeasible. Hence, the study of plant networks is limited primarily by the identification of plant network links. To overcome these limitations, I propose the concept and implementation of constructive networks.

The ‘ability’ of plants to change the structure of habitats, modulate resources available to other species, and influence the physical, chemical and biological conditions of the environment led to the development of the concepts of nurse plants, keystone plants, ecosystem engineers (Jones et al. 1994) and foundation species (Ellison et al. 2005) (see also (Lewontin 1983; McIntire and Fajardo 2014)). The formalization of these concepts further developed into the frameworks of integrated community (Lortie et al. 2004), niche construction theory (Odling-Smee et al. 1996) and contemporary niche theory (Chase and Leibold 2003).

Constructive networks build on and unify niche construction and complex network theories. Constructive networks are ensembles of different relationships and interaction types including plant–plant and species–environment interactions. They recognize that plants have manifold ecological functions and the environment shapes and is shaped by diverse organisms, primarily plants. Constructive networks integrate both plant–environment and plant–plant interactions, addressing the way in which plants influence the environment, other plants, and the interactions between the environment and other species. In a relational context, constructive networks consider that plants support or inhibit other plant species by physically (e.g. mosses living on tree bark), biochemically (e.g. fixing soil nitrogen) and ecologically (e.g. reducing heat and drought stress) shaping environmental conditions, species–environment or plant–plant interactions. In constructive networks, diverse plants change and create novel environmental conditions and co-produce, transform and share resources, thereby influencing the environment in constructive ways.

Associations between plant species (i.e. significantly higher or lower co-occurrence frequency than expected by chance) can be used as a proxy of plant facilitation links under certain circumstances. For instance, when facilitation mechanisms are known, such as in the well-studied cases of nurse plants in high-alpine and arid environments (Verdú and Valiente-Banuet 2008; Burns and Zotz 2010; Saiz et al. 2017), plant associations provide a reliable signal for inferring plant network links (Alcántara et al. 2019; Losapio et al. 2019). But plant facilitation goes beyond nurse plant systems and conspicuous vegetation patterns (Liancourt and Dolezal 2021).

**Figure 3:**
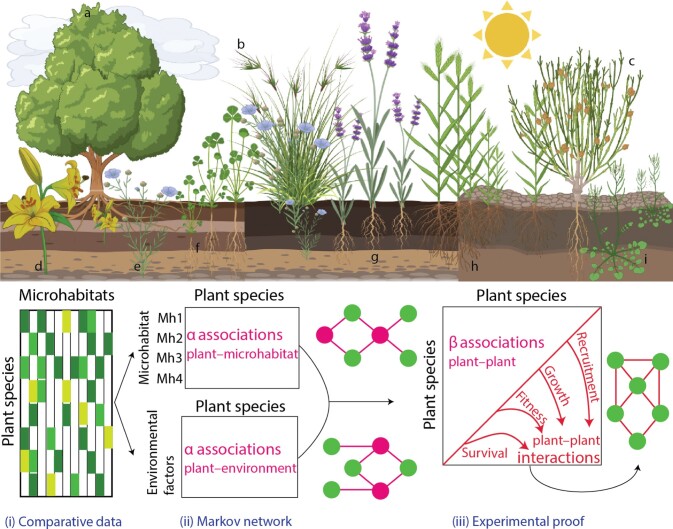
**(Top)** Ecosystem representing different plant species, some of them acting as foundation species (ash tree, a), keystone species (kangaroo grass, b) or nurse plants (*ephedra*, c). Microhabitats are characterized by plant communities creating different soils and microclimate conditions. Some plant species live only in association to foundation or keystone species, such as lily (d) or flax (e). Species such as barrelclover (f) grow worst with ash tree but better with kangaroo grass, while others like lavender (g) are not particularly influenced by neighbours. Finally, plant species like stiff brome (h) and hairy bittercress (i) are facilitated by nurse plant *ephedra*. **(Bottom)** Workflow prototype for implementing constructive networks with Markov networks. Comparative data of plant communities across microhabitats are the input matrix. Results of Markov networks will provide $\alpha$ and $\beta$ parameters, indicating plant–microhabitat and plant–plant associations, respectively. Different environmental factors such as soil and microclimate variables can also be used for addressing plant–environment interactions. Using $\alpha$ associations, one can build plant–microhabitat networks in which nodes and links represent plant species and microhabitats and their relationships, respectively. Then, one can build plant–plant association networks using $\beta$ parameters in Markov networks. These putative interactions shall be further confirmed empirically with additional and independent data on ecological mechanisms of plant–plant interactions, including facilitation or competition for recruitment, growth and reproduction.

Provided that co-occurrence is considered at the adequate spatial scale and multiple factors are taken into account, inferred statistical associations between plants from co-occurrence data may provide a signal for putative plant interaction links (Losapio et al. 2021b). Then, putative interactions should be further confirmed empirically with additional and independent data on plant recruitment, growth, survival, reproduction and fitness (Fig. 3). Notably, this way, one can also distinguish between the long-term ecological outcome of plant interactions (e.g. spatial displacement or spatial aggregation) from the plethora of underlying mechanisms (e.g. increase in water uptake or decrease in pollination).

Currently, the best way forward to resolve the question of how to infer plant–plant interactions from associations and build plant networks is given by statistical physics. I propose here to adopt a novel analytical model of Markov networks (Harris 2016). With Markov networks it is possible to make inference about the association matrix from co-occurrence data on the basis of conditional relationships among species (Azaele et al. 2010). In its canonical definition, a Markov network defines the relative probability of observing a pool of species as


$$p(\vec{y};\alpha ,\beta )\propto exp\left(\sum_{i}\alpha_{i}y_{i}+\sum_{ij}\beta_{ij}y_{i}y_{j}\right)$$

where $\alpha$ is the direct effect of environmental factors on each species *i*, and $\beta$ is the relative probability that target species *i* and neighbouring species *j* will co-occur, conditioned by species-specific environmental/microhabitat effects and after controlling for the other species in the network (Fig.3).

When a plant species *i* is particularly associated to a microhabitat or an environmental factor, then $\alpha_{i}>0$, while $\alpha_{i}<0$, if a plant species does not thrive in a microhabitat or is negatively affected by an environmental factor. Similarly, $\beta_{ij}<0$, if two plant species *i* and *j* are negatively associated with each other, otherwise $\beta_{ij}>0$, if two plant species are positively associated. The model can be generalized to any plant community with different plant species, microhabitats and environmental factors. Model coefficients $\alpha_i$ and $\beta_{ij}$ would be better estimated from abundance data rather than the presence/absence by parameterizing the Markov network model using Poisson or negative binomialdistributions.

**Figure 4:**
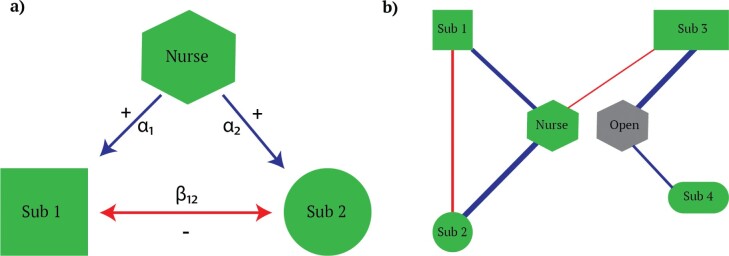
**a)** A small Markov network of one nurse plant species (‘Nurse’) and two other plant species (‘Sub 1’ and ‘Sub 2’). Arrows indicate positive (‘$+$’, blue) and negative (‘-’, red) associations, which point from the nurse to the subordinate for the $\alpha_i$ interaction coefficient, and point between subordinates in case of $\beta_{ij}$ interaction coefficient. Arrow size indicates association strength. **b)** A constructive network with different plant species and link types where nurse plants facilitate the occurrence of two plant species (Sub 2 and Sub 1), while excluding a third species (Sub 3) which thrives in open microhabitats. Meanwhile, putative competition is occurring among two ‘subordinate’ plant species. Line thickness is proportional to link weights.

These inferred parameters, which once again are statistical associations among plant species and are only putative of interaction outcome, shall be further compared to null models and can be used as links in constructive networks. A constructive network would contain the following two matrices (Fig.3): the first with species–environment relationships $\alpha_i$ and the second with species–species associations $\beta_{ij}$. These two matrices can be collated into a single multi-layer network (Fig.4). This way, species can have different links in terms of link types, such as species–environment relationships $\alpha_i$ and species–species associations $\beta_{ij}$, and with varying strength and directionality, that is, positive and negative links. As species–species associations are correlative and symmetrical, that is, $\beta_{ij}=\beta_{ji}$, there is just one link between two plant species.

**Figure 5:**
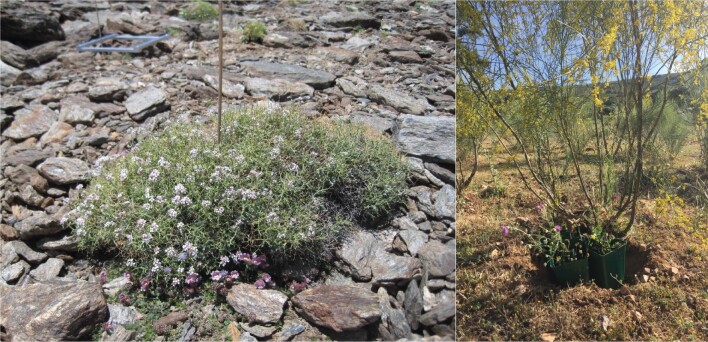
Example of plant systems where different plant species act as ecosystem engineers (left: *Hormathophylla spinosa* in high-alpine ecosystem) or nurse plants (right: *Retama sphaerocarpa* in Mediterranean woodland). Neighbouring plant species were either removed or added as experimental treatments to address the effects and mechanisms of plant facilitation and interference in plant–pollinator networks.

However, plant co-occurrence provides only a putative outcome or an indication of interactions, so statistical associations between plant species must be interpreted as hypotheses about the outcome of species interactions (Blanchet et al. 2020). Some of the main advantages of using Markov networks to infer species interactions instead of, for example, Gaussian Graphical models (which make use of partial correlation coefficients) include the possibility of (i) distinguishing between species–environment and species–species relationships, and (ii) addressing non-linear dependencies between species and the environment.

Identifying and proving plant–plant interactions requires empirical evidence. Associations inferred through Markov networks can be validated experimentally or through additional, independent comparative data – for instance, with additional experiments on ecological mechanisms involving recruitment, stress amelioration, pollination attractiveness or herbivory protection. The new independent data should be integrated into the ensemble of constructive networks. At the end, we would have a much smaller set of potential interactions to be proved as compared to screening and testing all possible interactions. This represents a feasible option for building more robust species interaction networks.

In the specific case of nurse plant system facilitation (Fig.4a), this Markov network model shall be adapted considering the microhabitat conditions created by nurse plants in conjunction with open areas (i.e. where nurse plants do not grow) as two distinct microhabitats, that is, the microhabitat/environment in the previous equation. In the resulting constructive network (Fig.4b), nurse plants, ‘subordinate’ species and open microhabitats are the nodes, the nurse– and open–subordinate interactions and subordinate–subordinate interactions are the links estimated by $\alpha_i$ and $\beta_{ij}$ coefficients, respectively. Then, model parameters $\alpha_i$ and $\beta_{ij}$ can be verified empirically by manipulating plant occurrence in different microhabitats or plant density in replacement series and then looking at plant performance and outcomes.

Finally, to understand how plant species influence and are influenced by the environment, plant communities can be coupled to environmental factors by means of dynamic models (Kéfi et al. 2012; Levine et al. 2017; Saavedra et al. 2017; Losapio et al. 2021b). A system of differential equations can be used to describe species–environment interactions including plant community dynamics and environment state variables. The community dynamics of plant *S* species, that is, changes in abundance/cover *N_i_* of plant species *i* over time *t*, in response to environmental conditions *k* can be described using inferred Markov network parameters as


$$\frac{dN_{i}}{dt}=N_{i}\left(r_{i}+\sum_{j=1}^{S}B_{ij}N_{j}\right)+f(A_{i},k)N_{i}$$

where ${f}(A_{i},{k})$ is the function describing the rescaled effect $\alpha_i$ of environmental factor on *k* plant species *i*, and *B_ij_* is the rescaled effect of plant species *j* on *i*.

The dynamics of the environment *E* representing changes in the environmental factors *k* can be described as


$$\frac{dE_{k}}{dt}=f(K)+f(\gamma_{ki})N_{i}$$

where ${f}({K})$ is the function describing the state of global environmental conditions *k* at local environmental scale, and ${f}(\gamma_{ki})$ is the function describing the effects $\gamma_{ki}$ of plant species on the environment. In this general form, one can parameterize $\gamma_{ki}$ according to all the various ways in which plant species influence the environment by altering energy, water, carbon and nutrient fluxes.

## Conclusions

In agreement with niche construction and complex network theories, I propose the notion of constructive networks. Constructive networks integrate fundamental biological processes with first principles of plant ecology. The unique properties of plants that make them producers of oxygen, organic matter and resources locate them at the core of mass, energy and information flows. Beyond their topological position within ecological networks, plants modify environmental conditions in ways that influence the same network at higher levels. Multiple mechanisms are known by which plants interact with each other, but many still remain to be discovered. Plant engineering and diversity effects are just two iconic examples of how plants construct environments where other species can thrive. However, much remains to be done in order to broaden and deepen our understanding of the multiple mechanisms by which plant change the environment bottom-up and have a direct or indirect influence on species and communities. Moving beyond models based on animal competition and top-down environmental control requires the development of novel analytical approaches and experiments. Identifying plant links is a difficult task limiting our understanding of plant–plant networks. This limitation can be overcome by integrating complexity theories with comparative data, statistical modelling and experimental field botany. Constructive networks can help us to identify the role of plant network features and ecological processes contributing to biodiversity maintenance and ecosystem functioning, and to anticipate the response of plant systems to global change.

## Sources of Funding

I acknowledge support from the Swiss National Science Foundation (grant number PZ00P3_202127).

## Contribution by Author

The author conceived and wrote the article.

## Acknowledgements

I would like to thank Prof. Dr. Christian Schöb for his initial comments, encouragement and inspiration. Thanks to Lilian Dutoit for helping with manuscript writing, editing and proofreading. I acknowledge the time and unpaid work that Associate Editor Anna Traveset and two anonymous reviewers put into reviewing an early version of this manuscript.

## Ethics declarations

The author declares no competing interests.

## Data availability

Data reporting number of papers published in different fields (Fig. 2) can be found in Supplementary Material.

## Supporting Information

The following additional information is available in the online version of this article – revdata.csv

## Supplementary Material

plad035_suppl_Supplementary_Material

## Notes

**Natural History and Conservation. Chief Editor:** F. Xavier Pico
